# Re-structuring lentiviral vectors to express genomic RNA via cap-dependent translation

**DOI:** 10.1016/j.omtm.2020.12.005

**Published:** 2020-12-15

**Authors:** John R. Counsell, Guillaume De Brabandere, Rajvinder Karda, Marc Moore, Antonio Greco, Alysha Bray, Juan Antinao Diaz, Dany P. Perocheau, Ulrike Mock, Simon N. Waddington

**Affiliations:** 1 Dubowitz Neuromuscular Centre, Molecular Neurosciences Section, Developmental Neurosciences Programme, UCL Great Ormond Street Institute of Child Health, 30 Guilford Street, London, UK; 2 NIHR Great Ormond Street Hospital Biomedical Research Centre, 30 Guilford Street, London WC1N 1EH, UK; 3 Gene Transfer Technology Group, Institute for Women’s Health, University College London, 86-96 Chenies Mews, London, UK; 4 MRC Antiviral Gene Therapy Research Unit, Faculty of Health Sciences, University of the Witswatersrand, Johannesburg, South Africa

## Abstract

Lentiviral (LV) vectors based on human immunodeficiency virus type I (HIV-1) package two copies of their single-stranded RNA into vector particles. Normally, this RNA genome is reverse transcribed into a double-stranded DNA provirus that integrates into the cell genome, providing permanent gene transfer and long-term expression. Integration-deficient LV vectors have been developed to reduce the frequency of genomic integration and thereby limit their persistence in dividing cells. Here, we describe optimization of a reverse-transcriptase-deficient LV vector, which enables direct translation of LV RNA genomes upon cell entry, for transient expression of vector payloads as mRNA without a DNA intermediate. We have engineered a novel LV genome arrangement in which HIV-1 sequences are removed from the 5′ end, to enable ribosomal entry from the 5′ 7-methylguanylate cap for efficient translation of the vector payload. We have shown that this LV-mediated mRNA delivery platform provides transient transgene expression *in vitro* and *in vivo*. This has a potential application in gene and cell therapy scenarios requiring temporary payload expression in cells and tissues that can be targeted with pseudotyped LV vectors.

## Graphical Abstract

**Figure:**
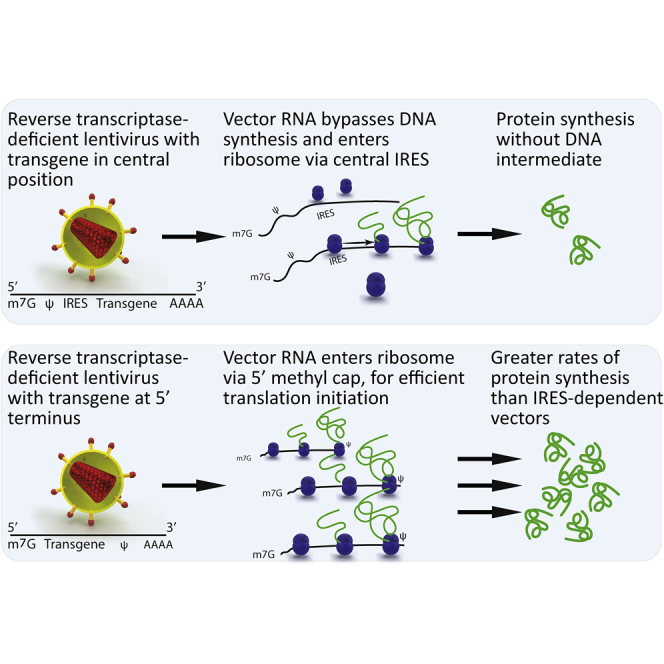

## Teaser

Here we describe the design and validation of a novel lentivirus-based mRNA delivery platform for delivery of therapeutic sequences to cells and tissues *in vivo* and *ex vivo*. The platform technology has potential use in the transient expression of engineered nucleases and vaccine development.

## Introduction

Lentiviral (LV) vectors based on human immunodeficiency virus type I (HIV-1) have been developed to deliver genetic material to a broad range of cell types. Integration-proficient LV (IPLV) vectors are the conventional form of LV technology, in which vector proviruses permanently integrate into the transduced cell genome. But these integration events sometimes occur within genes, which can dysregulate endogenous gene expression.^1^, ^2^, ^3^

To minimize integration events, integration-deficient LV (IDLV) vectors have been developed by mutating the HIV-1 integrase component of LV vectors to ensure that the majority of proviral DNA remains as extrachromosomal episomes. However, some chromosomal integration still occurs with IDLV technology, with 0.1%–1% of proviruses integrating into the genome.^4^ This is potentially undesirable when vector persistence has the potential to detrimentally affect recipient cells or when transgene expression needs to be controlled in a temporal manner.

Messenger RNA (mRNA) delivery offers a means to transiently express exogenous genes in a target cell, as the delivered mRNA remains extranuclear. Non-viral vectors have been developed for *in vivo* mRNA delivery, but tissue-specific targeting requires further optimization.^5^ A variety of retroviral vectors have been engineered for transient delivery of their single-stranded RNA (ssRNA) genomes for direct mRNA translation by mutating their reverse-transcriptase (RT) coding sequence.^6^ Additionally, viral vectors based on Sendai virus have been developed for *in vivo* delivery, but toxicity and limited efficacy preclude their clinical translation.^7^

HIV-1-based LV vectors offer a potential means to deliver mRNA to a wide range of cell types *in vivo* and *in vitro*, as they package their genomes in the form of ssRNA. In conventional LV vectors, the ssRNA genome is reverse-transcribed to give a double-stranded DNA (dsDNA) product, which then enters the nucleus. Recently it has been shown that HIV-1 RT can be engineered to prevent RT, thus permitting immediate translation of LV ssRNA genomes upon cell entry. However, this design remains inefficient, highlighted by low rates of efficacy in *ex vivo* cultured hematopoietic stem cells (HSCs).^5^ Further attempts to enhance LV vectors for mRNA delivery have included incorporation of the bacteriophage MS2-Coat protein into LV capsids and engineering the MS2 RNA stem loop into LV genomic RNA.^8^ This MS2-mediated RNA delivery vector showed strong potential for gene delivery *ex vivo* and *in vivo*, but clinical application of these chimeric LV-MS2 particles has yet to be reported.

The use of LV vectors for mRNA delivery has potential advantages for applications requiring transient gene expression in a specific cell type. This includes *in vivo* and *ex vivo* gene editing, where it would be desirable to exploit vesicular stomatitis virus G protein (VSV-G)-based transduction of target cell lines without long-term expression of gene-editing nucleases.^9^ Additionally, the possibility to target antigen-presenting cells (APCs) with LV vectors has been explored for vaccine development.^10^^,^^11^ Use of a transient mRNA delivery system in a LV context could offer a significant development in these areas of research, avoiding any issues related to persistence of IDLV or IPLV vector genomes.

Here, we describe the development of a novel HIV-1-based LV vector that has been engineered for direct expression of its ssRNA payload upon target cell entry. We have restructured and iteratively optimized the LV genome for this purpose by relocating all HIV-1 material to the 3′ untranslated region,^12^ meaning that ribosomal entry occurs at the 7-methylguanylate (m^7^G) 5′ cap of the vector RNA. We show that this 5′ cap-dependent LV (CDLV) vector significantly improves the efficiency of LV ssRNA translation, compared to previously developed technologies that require an internal ribosomal entry site (IRES) for translation.^5^ Furthermore, we demonstrate that CDLV technology can deliver transient gene expression to mouse liver *in vivo*, matching the expression of DNA-based IDLV genomes over a 24-h period. This introduces CDLV as a novel platform technology for potential use in transient treatment and manipulation of target cells *in vitro* and *in vivo*.

## Results

**Figure 1:**
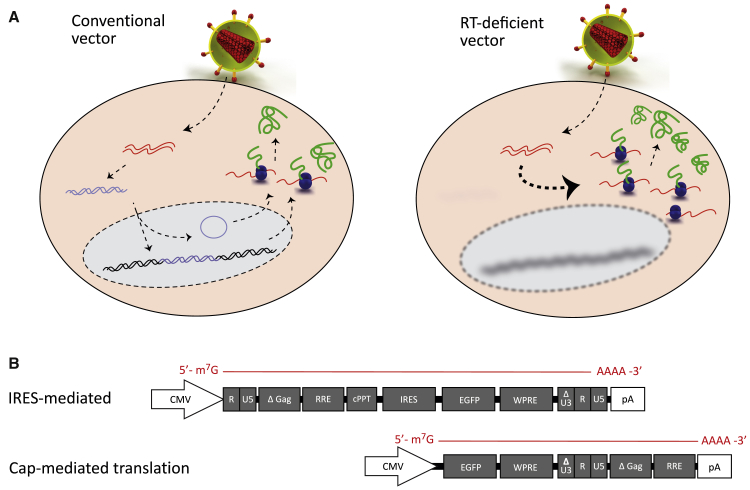
Schematic showing the life cycle of LV vectors designed for direct mRNA expression (A) In the left panel, a conventional LV transduces the cell and reverse transcribes its ssRNA into dsDNA, which then enters the nucleus to either integrate into the host genome or exist as a circular “episome.” The nuclear DNA can then use host machinery to drive transgene mRNA production and express the transgene protein. In the right panel, RT-deficient LV vectors contain a mutated RT enzyme, which means that packaged ssRNA is free to interact with cell ribosomes to express contents as mRNA. (B) Vector schematics show the structure of the DNA template used to generate vector genome RNA. White boxes indicate elements that are present in DNA alone, while gray boxes indicate the elements that are transcribed into the encapsidated RNA genomes. “IRES-mediated” mRNA expression has previously been reported, where the vector RNA genome contains an internal IRES, which allows translation of the downstream coding sequence (in this case, EGFP). With our novel “cap-mediated” mRNA expression system, the transgene coding sequence is positioned at the extreme 5′ terminus of vector ssRNA, meaning that translation initiation occurs from the 5′ m^7^G cap. Additionally, the vector lacks a HIV-1 primer binding site, further reducing any probability of the mutated RT binding its canonical target.

The aim of this investigation was to develop an LV-based vector that can efficiently deliver its genome as mRNA to target cells *in vivo* and *ex vivo*. It has previously been reported that the HIV-1 RT component of LV vectors can be mutated to remove its ability to convert RNA into DNA.^5^ This RT-deficient LV vector platform then achieves transgene expression in target cells without forming a DNA intermediate (Figure 1A).

### Engineering LV vector genomes to maximize translation of packaged ssRNA

In previous studies, RT-deficient vectors have achieved gene expression in a subset of target cells, but the expression level was insufficient to mediate targeted disruption of the human *CCR5* gene in HSCs.^5^ We hypothesized that this lack of efficacy was in part due to reliance on an IRES element to initiate translation, due to the presence of 1.5 kb wild-type HIV-1 DNA in the 5′ region of the vector RNA (Figure 1B).

In order to eliminate this problem, we restructured the vector genome by moving the 1.5 kb HIV-1 DNA downstream of the therapeutic transgene (Figure 1B). This configuration was designed to leave a Kozak consensus sequence^13^ at the extreme 5′ terminus of the vector RNA, which would be 5′ m^7^G cap capped during vector production.^14^ Additionally, the HIV-1 primer-binding site (PBS) was completely removed to eliminate canonical RT priming. Our expectation was that the 5′ m^7^G cap-mediated LV vector would produce a greater level of gene expression than the IRES-mediated configuration.

**Figure 2:**
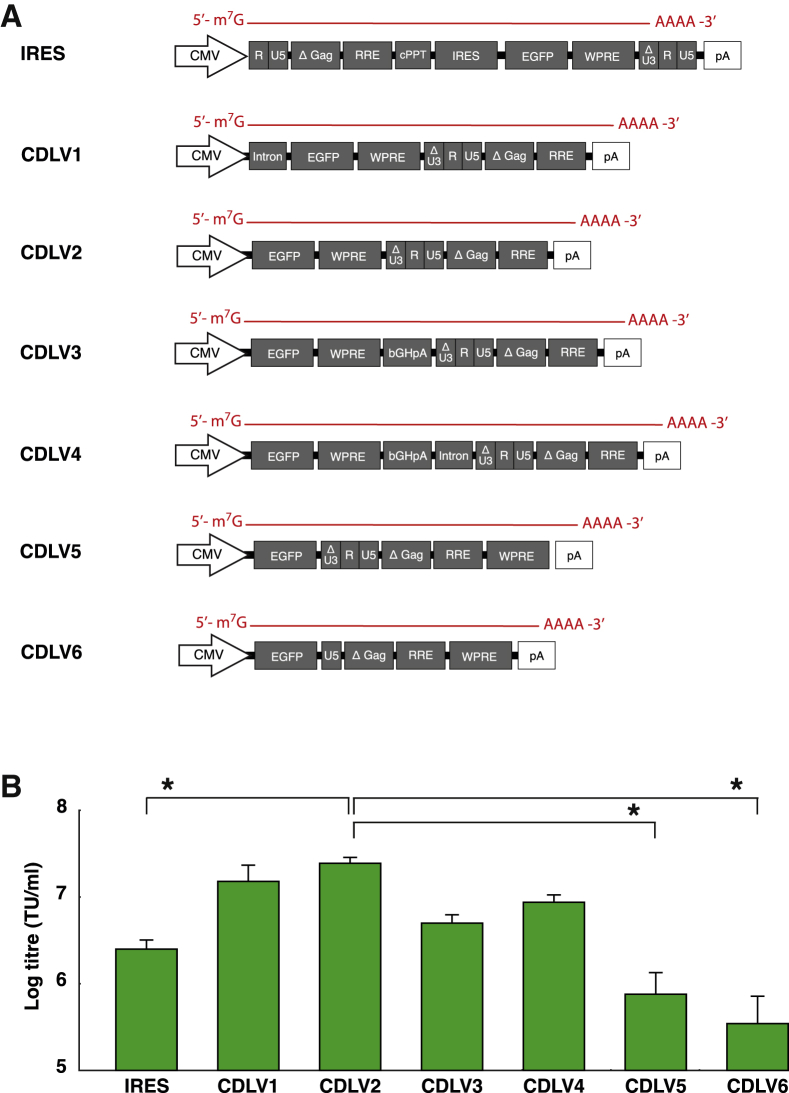
Identifying the optimal genome structure for CDLV translation (A) Six versions of CDLV (CDLV1-6) were developed, with designs aimed to enhance RNA processing for optimal packaging and translation upon cell entry. The schematics represent the structure of LV vector RNA genomes that would be packaged into vector particles. LTR, HIV-1 long terminal repeat; Δ Gag, truncated and inactive HIV-1 gag gene, including Ψ packaging sequence; RRE, HIV-1 rev response element; cPPT, HIV-1 central polypurine tract; IRES, internal ribosome entry site of the encephalomyocarditis virus; EGFP, enhanced green fluorescent protein; WPRE, woodchuck-hepatitis virus post-transcriptional regulatory element; Intron, chimera between introns from human β-globin and immunoglobulin heavy chain genes; bGHpA, bovine growth hormone polyadenylation sequence; U5, U5 domain of the HIV-1 LTR. (B) Comparison of functional titers gained from each of the ssRNA genomes. Titers were determined by the percentage of EGFP^+^ cells in transduced cells, indicating transduction units per milliliter (TU/mL). Statistical comparison of the titers was made by Kruskal-Wallis test with Dunn’s posthoc analysis (∗p < 0.001). Averages were calculated from 4 experimental replicates in each case. Error bars indicate standard error of mean (SEM).

We engineered six iterations of the cap-mediated “CDLV” vector, with the aim of identifying a configuration that could exceed IRES-based LV expression (Figure 2A). In each case, the depicted vector RNA is derived from a plasmid using the cytomegalovirus (CMV) promoter to transcribe vector genome RNA. Each variant contained variable combinations of non-coding domains of the transcribed region. Version 1 (CDLV1) was designed to include a chimeric intron (fusion of introns from human β-globin and immunoglobulin heavy chain genes), based on the expectation that the presence of splice sites at the 5′ end would enhance nuclear export of the RNA.^15^^,^^16^ CDLV2 was identical to version CDLV1, but for omission of the chimeric intron. CDLV3 included a poly(A) motif upstream of the 3′ long terminal repeat (LTR) to enhance LV ssRNA expression,^5^ while CDLV4 additionally contained an intron downstream of the poly(A), to minimize premature termination during vector production.^17^ Finally, CDLV5 and CDLV6 aimed to promote readthrough of the 3′ LTR by relocating woodchuck-hepatitis virus post-transcriptional regulatory element (WPRE) to the extreme 3′ terminus^18^ and deleting portions of the U3-R domains that contain HIV-1 poly(A) motifs.

In each case, vectors were designed to deliver an enhanced green fluorescent protein (EGFP) RNA payload. Vector titers were determined by transduction of HEK293T cells and quantifying the number of EGFP-positive (EGFP^+^) cells by flow cytometry. This comparison revealed that CDLV version 2 yielded titers one order of magnitude greater than the IRES-based vector (p < 0.001; Figure 2B). This structure was therefore taken forward for further characterization of the technology.

### Cap-dependent translation provides greater gene expression than IRES-dependent translation

**Figure 3:**
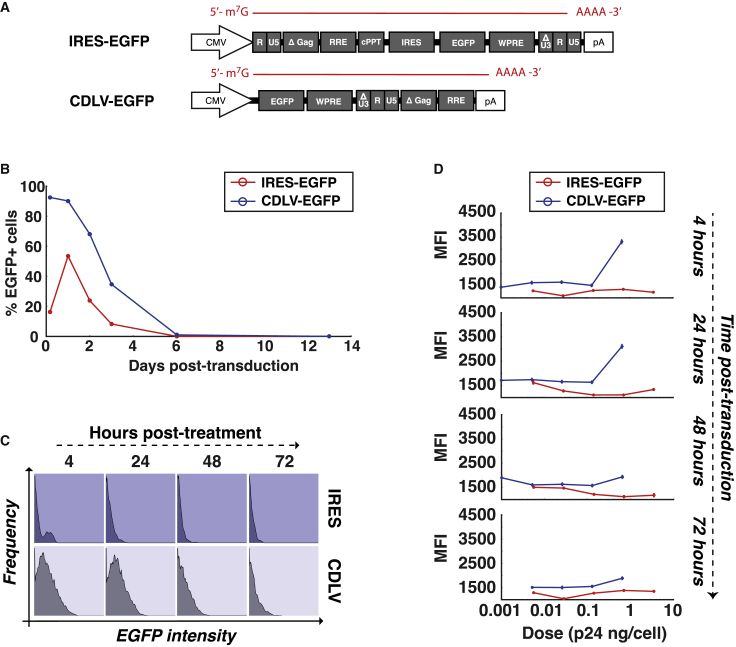
Comparison of CDLV transduction kinetics versus IRES-based RT-deficient vectors (A) IRES-EGFP and CDLV-EGFP vectors were produced using the depicted genomes. (B) HEK293T cells were transduced with the vectors. Longitudinal analysis of EGFP expression shows CDLV vector-expression profile compared versus the IRES-based system over the course of the experiment (p = 0.0196 by t test). (C) Flow cytometry plots show EGFP mean fluorescence intensity (MFI) per transduced cell, comparing CDLV-EGFP expression versus IRES-EGFP. (D) Vectors dosed by p24 capsid protein mass allow crude estimation of the number of vector capsids required to achieve optimal gene expression. This analysis demonstrates that 1 ng p24 per cell is necessary to achieve optimal expression intensity with CDLV (approximately 1 × 10^7^ LV particles).

The transduction kinetics of CDLV version 2 were compared in greater detail versus the IRES-mediated system. HEK293T cells were transduced with an equal dose of either CDLV-EGFP or IRES-EGFP (Figure 3A). Longitudinal analysis of EGFP expression showed that the CDLV vector produced a greater number of EGFP^+^ cells than IRES-based LV throughout the course of the experiment (p = 0.0196), with both vectors achieving peak expression by 24 h and dropping to minimal levels by day 6 (Figure 3B). EGFP fluorescence intensity was also quantified at each time point, with CDLV expression exceeding that of IRES-based vectors up to 72-h post-transduction (p < 0.001; Figure 3C).

Additionally, in order to clarify that these expression values were being derived from enhanced expression, rather than excess physical particle mass, dose response profiles were also compared in investigations in which vectors were dosed by capsid mass. These investigations showed that the CDLV vector gave the greatest level of EGFP expression at 4 h and 24 h post-transduction, when administered at a dose of 0.64 ng p24/cell. The IRES-based vector was unable to match this level, even when administered to cells at higher doses of 0.7 ng p24/cell and 3.5 ng p24/cell (Figure 3D). The intensity of EGFP expression from IRES vectors was not enhanced at any of the tested doses, indicating that much higher doses would be needed to accumulate equivalent EGFP levels as those observed for CDLV.

### CDLV vectors provide a transient burst of gene expression *in vitro*

**Figure 4:**
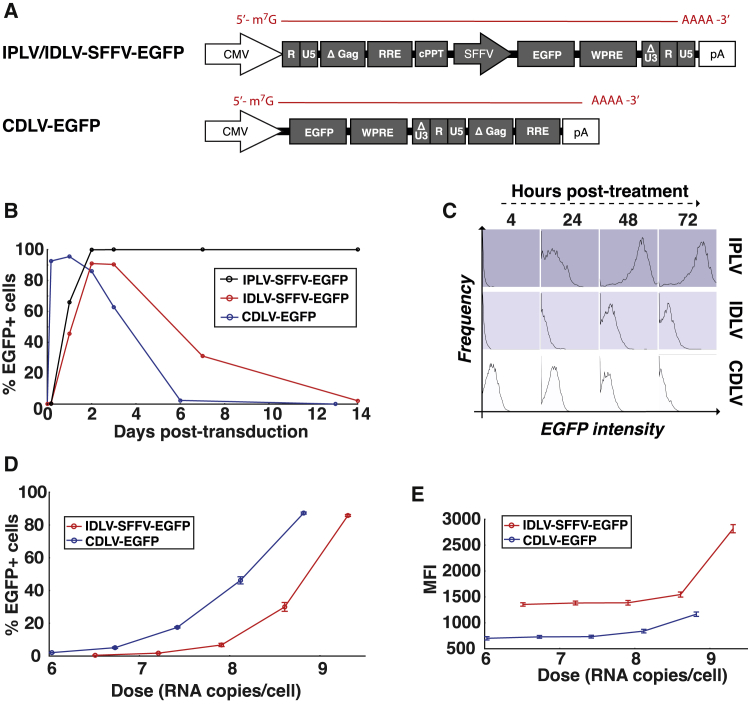
Comparison of CDLV transduction kinetics to conventional IDLV and IPLV vectors (A) Integration deficient (IDLV-SFFV-EGFP) and integration-proficient (IPLV-SFFV-EGFP) LV vectors containing an SFFV-EGFP expression cassette were compared to a CDLV-EGFP vector. (B) HEK293T cells transduced and the percentage of EGFP^+^ cells was tracked for 2 weeks to monitor expression longevity. (C) The intensity of EGFP expression gained from each vector is shown for the first 72 h after transduction. (D) The percentage of EGFP^+^ cells is compared for CDLV-EGFP at 24-h post-transduction and IDLV-SFFV-EGFP at 72-h post-transduction (the relative timing of peak expression for each vector platform; p = 0.15 by t test). (E) The same dataset shown in (D) was analyzed for EGFP fluorescence (MFI), to compare the strength of EGFP expression per transduced cell (p = 0.005 by t test) (error bars represent SEM).

After demonstrating the potential advantages of CDLV as an mRNA delivery platform, we set out to investigate how its longitudinal expression profile compares to DNA-based gene delivery systems. In this experiment, EGFP was again employed as a transgene, driven by the spleen focus-forming virus (SFFV) promoter in the DNA-based IPLV and IDLV vectors (Figure 4A).

CDLV-EGFP was delivered to HEK293T cells at a multiplicity of infection (MOI) of 41 EGFP-forming units per cell (EFU/cell), while IPLV-SFFV-EGFP and IDLV-SFFV-EGFP were delivered at doses of 10 EFU/cell. As expected, CDLV produced a transient expression profile, with peak expression occurring around 24 h post-transduction, matching the longitudinal profile seen with previous retrovirus-based mRNA delivery platforms.^5^^,^^19^^,^^20^ whereas IPLV and IDLV vectors peaked at around 48 h post-transduction, with the IDLV profile falling to 2.2% EGFP^+^ cells by day 14 (Figure 4B). Analysis of mean fluorescence intensity again showed that CDLV expression appeared sooner than IDLV and IPLV, with CDLV expression level remaining detectable throughout the initial 72 h post-transduction (Figure 4C). Quantification of vector DNA in the transduced cells did not reveal the presence of proviruses in any of the CDLV-transduced cells (data not shown), confirming that the RT mutation has eliminated RT functionality.^5^

Data presented in Figures 4B and 4C appear to show that CDLV expression peaks at 24 h post-transduction, whereas IDLV expression peaks at 72 h. To compare their relative gene-expression efficacy at these time-points, we titered CDLV and IPLV vectors by RNA genome copy number and administered to HEK293T cells at known vector genome doses, with EGFP expression measured at their respective peak time points. This investigation revealed that CDLV produced a similar number of EGFP^+^ cells to IDLV, even when administered at a lower RNA copy number (p = 0.15; Figure 4D). However, when examining the intensity of EGFP expression in the transduced cells, it was seen that IDLV treatment produced a greater level of expression per administered RNA genome (Figure 4E; p = 0.005), likely due to the use of a strong SFFV promoter in the IDLV format, leading to mRNA abundance.

### LV vectors can express ssRNA payloads in mouse liver *in vivo*

LV vectors are commonly pseudotyped with VSV-G, a glycoprotein that confers broad tissue tropism by targeting the low-density lipoprotein receptor (LDLR) for cell entry.^21^ VSV-G-pseudotyped LV vectors are particularly effective for *in vivo* liver transduction.^22^ We investigated the effectiveness of our engineered CDLV vector for gene transfer to neonatal mouse liver *in vivo*, comparing its longitudinal expression profile to a conventional IDLV vector.

**Figure 5:**
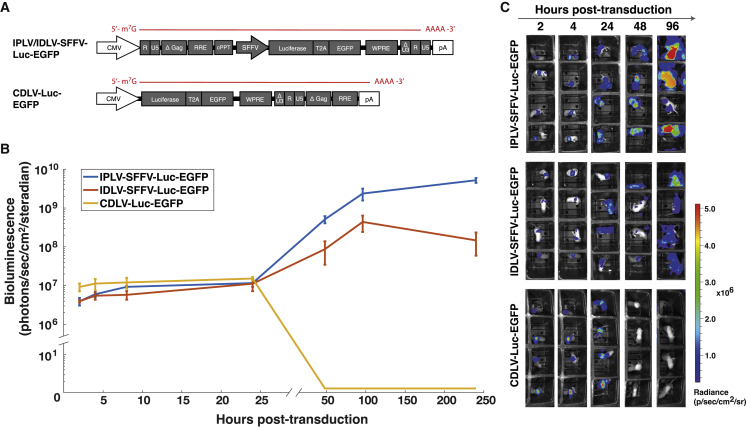
Tracking the *in vivo* expression kinetics of CDLV compared to DNA-based LV vectors (A) IDLV or IPLV vectors were produced containing a bicistronic luciferase-EGFP expression cassette, driven by the SFFV promoter (IPLV/IDLV-SFFV-Luc-EGFP). A CDLV vector was additionally produced containing the same “Luc-EGFP” coding sequence (CDLV-Luc-EGFP). (B) *In* vivo bioluminescence was imaged at various time-points for 10 days (240 h) after vector injection, to track the onset and persistence of transgene expression. Data are expressed as photons per second per cm^2^ per steradian (n = 4 mice per group, error bars represent SEM). (C) The associated heatmaps for each mouse indicate bioluminescence signal intensity over the initial 96 h of the experiment.

We used a bicistronic transgene expressing luciferase and EGFP, separated by a 2A cleavage peptide derived from thosea asigna virus.^23^ This “Luc-EGFP” reporter was packaged into IPLV and IDLV vectors driven by the SFFV promoter (IPLV-SFFV-Luc-EGFP and IDLV-SFFV-Luc-EGFP, respectively). The Luc-EGFP transgene was additionally packaged into a CDLV vector (CDLV-Luc-EGFP; Figure 5A).

Neonatal outbred mice received intravenous injections of each vector on the day of birth and bioluminescent imaging began 2 h after vector administration and continued for 10 days (Figures 5B and 5C). Vector doses were calculated based on ssRNA titers, with IPLV being delivered at 1 × 10^13^ viral genomes per milliliter (vg/mL), IDLV delivered at 5 × 10^12^ vg/mL, and CDLV delivered at 4 × 10^11^ vg/mL. As expected, IPLV vector expression was detectable at early stages post-transduction and expression intensity continued to increase throughout the course of the investigation. IDLV vectors showed reduction of bioluminescent signal after 96 h post-transduction, indicating reduced persistence in transduced liver. CDLV vector expression was detectable by 2 h post-injection, with its expression appearing to peak at 24 h before falling below the limit of detection thereafter.

## Discussion

LV vectors are effective gene transfer agents, with an ability to transduce a variety of cell types *in vitro* and *in vivo*. This has led to their application in a number of gene and cell therapies, particularly in circumstances where transgene capacity precludes use of AAV vectors or cell targeting is suboptimal with non-viral vector technologies. Their ability to permanently integrate their DNA into dividing and non-dividing cells has made them a valuable tool in stem cell therapies, as modified cells will retain the therapeutic payload throughout cell division.

Here, we show that HIV-1-based LV vectors can be used as transient mRNA delivery vehicles by engineering the RT and RNA genome to promote translation of the transgene from the 5′ m^7^G cap, which we show delivers immediate but transient mRNA expression both *in vitro* and *in vivo*.

The findings of our work are of potential relevance to retrovirology and the timing of genomic RNA release. A number of mechanisms have been proposed for this aspect of transduction, one of which is that capsid disassembly occurs during the early stages of RT.^24^ Given that RT is dysfunctional in our system, but EGFP is clearly detectable soon after cell entry, this suggests that vector RNA is released immediately after cell entry, irrespective of RT.

During the development of the CDLV system, we engineered a variety of gene-expression cassettes to modify the packaging and expression efficiency of vector ssRNA. Version 2 was taken forward to further investigations, given that it produced the most efficient EGFP expression. We demonstrated that this structure maximizes *in vitro* expression compared to the IRES-based version in a conventional 3^rd^ generation LV backbone. Further, we also demonstrated that CDLV can transduce cells *in vitro* with comparable frequency to non-integrating and integrating 3^rd^ generation LV vectors, but with complete transiency. Additionally, we showed that CDLV could achieve greater transduction frequency than IDLV, albeit with weaker expression levels per transduced cell.

Finally, given efficient liver targeting of VSV-G-pseudotyped LV vectors,^25^^,^^26^ we investigated the ability of CDLV to deliver transient gene expression to hepatocytes *in vivo*, employing a luciferase reporter to track live vector expression kinetics *in vivo*. This study showed that CDLV could indeed provide short-term transgene expression *in vivo*, demonstrating a similar profile to that obtained *in vitro*. Luciferase expression from the CDLV platform was comparable to IPLV and IDLV 3^rd^ generation vectors during the early stages after injection, despite the 3^rd^ generation vectors being delivered at higher doses. Additionally, it is important to note that in all experiments 3^rd^ generation IPLV and IDLV vectors were driven by a strong SFFV promoter, which will turn over high levels of mRNA in hepatocytes and HEK293T cells. In gene therapy, promoters weaker than SFFV are usually preferred, particularly in a LV context, due to potential safety concerns.^27^ Therefore, the gene-expression profiles that we have detected from CDLV technology are arguably comparable to clinically relevant LV vector cassettes. However, validation of the *in vivo* scalability of our platform will require further studies beyond neonatal mice, given that larger animal models may not be easily transduced *in vivo* with LV vectors.

The ability to deliver LV genomes as mRNA *in vitro* and *in vivo* brings some potential benefits in gene and cell therapy. LV vectors have a relatively large packaging capacity, able to package the mRNA of the majority of human genes.^28^ Therefore, CDLV technology has the potential to express large therapeutic transcripts transiently with high efficiency. This presents an advantage over non-viral gene transfer technologies, as transfection efficiency is known to reduce in correlation with increasing nucleic acid length.^29^^,^^30^ Indeed, LV vector gene transfer efficacy is also known to reduce in correlation with payload size, but it is noteworthy that this effect is thought to be limited primarily by inefficient RT of large payloads, rather than ssRNA packaging,^31^ which suggests that CDLV payload tolerance could be even higher than that of conventional LV vectors.

Perhaps one of the most interesting avenues for exploiting CDLV technology would be delivery of genome-editing nucleases. Cas9 nuclease mRNA has been used for *in vitro* and *in vivo* applications, although novel mRNA delivery strategies are continually being explored to enhance gene transfer efficiency.^32^ LV delivery of mRNA holds significant advantages here, as it has been shown over the past 20 years that LV vectors can be pseudotyped with a range of glycoproteins for targeted transduction of a wide range of cell types.^25^ Additionally, the packaging capacity of LV vectors means that Cas9 expression transcripts can be easily modified to include any of the recently developed modules (e.g., base editors,^33^^,^^34^ transcriptional activators,^35^ and repressors^36^), without compromising packaging ability.

An additional platform that could benefit from CDLV technology is in vaccinology, where researchers are developing methods to express antigens in APCs *in situ.*^10^ LV vectors have been investigated extensively for this purpose and it has been shown that a lack of pre-existing immunity allows repeated administrations.^11^ However, a potential limitation of LV use for APC transduction is the expression of SAMHD1 (SAM and HD domain-containing deoxynucleoside triphosphate triphosphohydrolase 1) in these immune cells, which lowers intracellular dNTP pools and reduces transduction efficiency by restricting the activity of RT.^37^^,^^38^ Therefore, given that CDLV technology is not dependent on RT for mediating expression, our platform technology could provide an advantage in this area of gene therapy.

In summary, we report design and development of a novel LV gene structure that enhances translation of packaged genomic mRNA immediately upon cell entry, with limited duration. We have shown that this novel CDLV vector can be used for gene expression both *in vitro* and *in vivo*, for potential applications in gene therapy.

## Materials and methods

### Generation of plasmid constructs

All plasmid constructs were made using standard molecular cloning procedures and PCR-mediated deletion of plasmid sequences.^39^ In cases where novel sequences or HIV-1 sequence deletions were incorporated, synthetic DNA fragments were designed and ordered as gBlocks (Integrated DNA Technologies). Detailed plasmid information is available upon request.

### Production of LV vectors

LV vectors were produced as described previously.^12^ Briefly, 1.8 × 10^7^ HEK293T cells were plated per 15 cm sterile culture dish and transfected with the following components: 40 μg of the relevant transfer plasmid, 20 μg of pMDLg.RRE, 10 μg of pRSV-Rev, and 10 μg of pMDG.2 (all plasmids produced by PlasmidFactory). Additionally, 10 μg of pCMV-Tat (kindly provided by Professor Axel Schambach from Hannover Medical School^40^) was supplemented for enhanced vector titers. The plasmid mixtures were added to 5 mL Opti-MEM and filtered through 220 nm sterile filter units. Filtered DNA was combined with 5 mL Opti-MEM (Life Tech/GE) containing 2 μM polyethylenamine (PEI, Sigma). The resulting 10 mL mixture was incubated at room temperature for 10 min before addition to HEK293T cells. After 4 h, the transfection mixture was replaced with fresh culture medium. Virus supernatant was collected at 48 h and 72 h post-transfection. After each harvest, the collected medium was filtered through a cellulose acetate membrane (0.45 mm pore). LV harvests were combined before concentration by ultracentrifugation. Briefly, viruses were placed in polyallomer centrifuge tubes (Beckman Coulter) and centrifuged for 2 h at 90,000 × *g* at 4°C in a Sorvall Discovery 90SE Centrifuge. Following centrifugation, the supernatant was removed and pellet recovered in 200 μL Opti-MEM.

### Titration of LV vectors

Vector titration by flow cytometry was performed as follows: 1 × 10^5^ HEK293T cells were plated into each well of a 6-well plate and transduced with a dose escalation of concentrated LV. For IDLV and IPLV vectors, EGFP measurements were made at 72 h post-transduction, whereas CDLV analysis was performed at 16 h post-transduction. Titers were calculated based on cell populations in the range of 5%–30% EGFP^+^, as described previously.^12^ EGFP^+^ cells were identified as described below in the Detection of eGFP expression in transduced cells section.

Vector titration by p24 capsid antigen was performed as follows: a p24 ELISA kit (Clontech product 632200) was used to determine the LV vector capsid number, according to the kit manufacturer’s calculations, where 1 ng p24 is equivalent to ∼1.25 × 10^7^ LV particles.

Vector titration by ssRNA genome copies was performed as follows: ssRNA genome copies were quantified using a qRT-PCR titration kit (Clontech product 631235). In brief, vector RNA was initially extracted from viral particles using spin columns and quantified by nanodrop. The vector RNA copy number was then calculated using an qRT-PCR assay targeting the HIV-1 RNA packaging sequence and extrapolating the absolute value from a standard curve of known vector genome copy numbers.

### Detection of EGFP expression in transduced cells

Unless stated otherwise, 100,000 cells were analyzed for EGFP expression in a BD FACSArray Bioanalyzer. During analysis, live cells were determined by gating forward light scatter versus side scatter and isolating the relevant population. EGFP^+^ cells were determined by plotting EGFP fluorescence (detected using a 530/30 nm bandpass filter) versus emission from the yellow channel (detected using a 575/26 band-pass filter) to compensate for auto-fluorescence. Non-transduced controls were used to gate background expression in each channel. All flow cytometry data were analyzed by FlowJo software version 9.3.1 (Tree Star).

### Animal procedures

For *in vivo* investigations, outbred CD1 mice (Charles River), were time mated to produce neonatal animals. At postnatal day 1, non-randomized neonates were subjected to brief hypothermic anesthesia and intravenously injected with LV vectors via the superficial temporal vein. Experimental groups were blinded during the course of *in vivo* investigations. Experiments were carried out under United Kingdom Home Office regulations and approved by the ethical review committee of University College London.

### Longitudinal tracking of vector expression *in vivo*

To monitor LTR1 bioluminescence *in vivo*, we administered 40 μL of the relevant luciferase expression vector intravenously to 1-day-old neonatal CD-1 mice. Vector doses were based on ssRNA titration results. Doses were calculated as 1 × 10^13^ vg/mL for IPLV, 5 × 10^12^ vg/mL for IDLV, and 4 × 10^11^ vg/mL for CDLV. Images and bioluminescence data were gathered continually for 10 days, as described previously,^41^ by intraperitoneal injection with firefly D-luciferin (150 mg/kg) and imaged after 5 min with a cooled charge-coupled device (CCD) camera (IVIS Lumina II, PerkinElmer). Detection of bioluminescence in the liver was performed using the auto region of interest (ROI) quantification function in Living Image 4.4 (PerkinElmer). Signal intensities were expressed as photons per second per centimeter^2^ per steradian.

### Statistical analysis

All statistical analyses were carried out using MATLAB 2015a. A Kruskal-Wallis test with Dunn’s posthoc analysis was used to compare vector titers. Student’s t test was used to compare mean fluorescence intensities and EGFP values, with dose-response profiles in Figure 4 compared according to trapezoidal area under the curve. *In vitro* experiments in cultured cells were performed in 4 experimental replicates. Mouse sample sizes were limited to 4 animals per experimental group for *in vivo* investigations.

### Data and materials availability

All plasmids are available under MTA agreement with UCL Business (mta@uclb.com).
